# Association of Indoor Temperature Level and Mental Health Among Community-Dwelling Older Adults: Systematic Review

**DOI:** 10.2196/78257

**Published:** 2025-11-27

**Authors:** Min Kyung Park, Bada Kang, Dahye Hong, Seolah Yoon, Minsub Kim, Yubeen Kim, Zhao Ni, Eleanor S McConnell

**Affiliations:** 1 Mo-Im Kim Nursing Research Institute, Yonsei University College of Nursing, 50-1 Yonsei-ro, Seodaemun-gu, Seoul, 03722, Republic of Korea, 82 2-2228-3283; 2 College of Nursing and Brain Korea 21 FOUR Project, Yonsei University, Seoul, Republic of Korea; 3 Department of Nursing, Yonsei University Graduate School, Seoul, Republic of Korea; 4 School of Nursing, Yale University, Orange, CT, United States; 5 School of Nursing, Duke University, Durham, NC, United States; 6 Geriatric Research, Education, and Clinical Center (GRECC), Durham VA Health Care System, Durham, NC, United States

**Keywords:** emotional, housing, climate change, sleep-wake disorder, social participation

## Abstract

**Background:**

The association between indoor temperature level and mental health is becoming increasingly important as climate change leads to extreme temperature fluctuations. Older adults are particularly vulnerable to indoor temperature changes because of their diminished ability to regulate body temperature and extended time spent indoors.

**Objective:**

This study aims to examine the association between indoor temperature levels and mental health outcomes among community-dwelling older adults, aiming to provide essential evidence to support the development of interventions and policy strategies to improve their mental health.

**Methods:**

In this systematic review, we conducted a comprehensive search of 7 electronic databases (MEDLINE, Embase, Cochrane Library, CINAHL, PsycINFO, Google Scholar, and ProQuest) on April 4, 2024, without restrictions on language or publication date. The National Institutes of Health quality assessment tool for observational cohorts and cross-sectional studies was used to evaluate the methodological quality of the included literature.

**Results:**

Of the 2328 studies identified, 15 met the inclusion criteria. The majority (8/15, 53%) were conducted in Asia, followed by Europe (4/15, 27%) and 1 study each in Australia (7%), Egypt (7%), and the United States (7%). Mental health outcomes associated with indoor temperature exposure were categorized into four groups: (1) sleep problems, including insomnia; (2) emotional problems, such as emotional distress and negative mood; (3) social interaction problems, such as social exclusion and low social participation; and (4) other mental health issues, including anxiety, agitation, and annoyance. Sleep problems were the most frequently reported mental health outcome related to indoor temperature levels (9/15, 60%). Older adults living in substandard housing conditions, facing economic difficulties, and residing in urban areas were vulnerable to exposure to uncomfortable indoor temperatures because of housing-related risks, such as low energy efficiency, inadequate heating or cooling, and limited access to green spaces.

**Conclusions:**

The findings highlight the need to develop evidence-based guidelines to improve mental health by managing indoor temperature levels in the community. Improving housing conditions through policy support could enhance the mental health of community-dwelling older adults.

## Introduction

Global temperature is rapidly increasing as a result of climate change [1]. The Lancet Countdown, an international collaboration monitoring the health impacts of climate change, warns that the unequal health impacts of climate change will worsen without immediate intervention [2,3]. As climate change leads to more frequent and intense extreme temperatures [1], interest is growing in studies on health outcomes associated with temperature level [4]. To better adapt to environmental changes caused by climate change, communities need to understand how to protect themselves from exposure to uncomfortable temperatures [5].

Temperature is closely linked to mental health, with growing evidence demonstrating that exposure to extreme temperatures is associated with increased hospital admissions and emergency department visits related to adverse mental health outcomes such as affective disorders, insomnia, anxiety, depressive disorders, schizophrenia, and organic mental disorders [6-8]. Indoor temperature may be evaluated by both objective measurements and subjective perceptions of indoor comfort [9], which is crucial given that recent studies have found that exposures measured by a single approach are significantly associated with negative mental health outcomes.

Determining an appropriate indoor temperature is complex as it depends not only on environmental and structural factors, such as regional climate and housing conditions [10,11], but also on individual response to temperature levels. Aging further complicates this issue, as older adults experience a diminished ability to perceive and regulate body temperature, increasing their vulnerability to the adverse effects of exposure to both high and low temperatures [12]. In addition, older adults are more susceptible to inappropriate indoor temperature exposure, because they tend to spend more time indoors than do younger populations [9,13]. A recent study found that community-dwelling older adults spend up to 85% of their time indoors, whether at home or in other settings [14]. Accordingly, a growing number of studies have examined the association between indoor temperature levels and mental health in older adults [15-17]. However, without synthesizing this fragmented evidence, it remains difficult to determine which mental health outcomes are most frequently reported in association with indoor temperature or to identify which subgroups of older adults are most vulnerable. A systematic review can integrate these findings to enhance our understanding of the associations between indoor temperature, individual vulnerabilities, and mental health outcomes, essential for informing targeted interventions and policy development in the context of aging populations and climate change.

In this systematic review, we aim to investigate the association between indoor temperature levels and mental health—encompassing both clinical disorders and nonclinical outcomes—in community-dwelling older adults, addressing the following questions:

1. What characteristics of indoor temperature exposure are related to mental health outcomes in older adults?
2. Which mental health outcomes are related to indoor temperature levels in older adults?
3. What factors make older adults vulnerable to indoor temperature levels?

## Methods

### Design

This study is a systematic review. The reporting of this review follows the Preferred Reporting Items for Systematic Reviews and Meta-Analyses (PRISMA) 2020 guidelines (Checklist 1) [18]. The protocol was registered in the International Prospective Register of Systematic Reviews (protocol: CRD42024536215). Owing to the use of previously published studies and publicly available anonymized data, ethical approval was not required.

### Search Strategy

A systematic search was conducted on 7 electronic databases with the assistance of experienced medical librarians: MEDLINE, Embase, Cochrane Library, CINAHL, PsycINFO, Google Scholar, and ProQuest. In addition, the bibliographies of the screened studies were manually searched. Three categories of concepts were used individually and in combination: (1) population (older adults), (2) exposure (indoor temperature), and (3) outcome (mental health). The database literature search was conducted on April 4, 2024, with no restrictions on publication dates or language. Non-English studies were screened using machine translation tools and cross-checked among the reviewers. The detailed search strategy for each database is provided in Table S1 in Multimedia Appendix 1.

### Eligibility Criteria

The inclusion and exclusion criteria are presented in Textbox 1.

Textbox 1. **Inclusion and exclusion criteria for study selection.**

**Inclusion criteria**

- Population: older adults aged ≥60 years.
- Exposure: indoor temperature measurement
- Outcome: mental health outcomes related to indoor temperature [19,20], including *Diagnostic and Statistical Manual of Mental Disorders*, Fifth Edition (DSM-5)-defined mental illnesses (eg, insomnia, mood disorders, and delirium) and nonclinical states (eg, annoyance, social isolation, and loneliness).
- Setting: community settings including homes and nonhospital residential environments (eg, geriatric and nursing homes).
- Study design: quantitative research, including cross-sectional and longitudinal studies.
- Language: no restrictions

**Exclusion criteria**

- Population: Studies not reporting separate results for older adults.
- Exposure: studies combining indoor and outdoor temperature without separate indoor effects.
- Outcome: studies without mental health–related outcomes.
- Setting: Hospital settings or nonresidential environments.
- Study design: protocol papers without data and studies lacking methodological reporting (eg, editorials and commentaries).
- Language: not applicable.

### Study Selection

The results of the literature search were exported from the databases into EndNote 20 reference management software (Clarivate Analytics) for organization and duplicate removal. The titles and abstracts of the retrieved articles were independently screened by 5 authors (MKP, DH, SY, YK, and MK), with all studies undergoing independent double review. For potentially relevant articles, 3 authors (MKP, DH, and SY) conducted full-text assessments independently in a double-review format. At each stage of the review process, any disagreements or discrepancies were resolved through discussion among the 3 authors until consensus was achieved.

### Data Extraction and Synthesis

Relevant data from eligible studies were extracted into a Microsoft Excel spreadsheet. The same 5 authors extracted data from the selected studies for analysis. One reviewer (MKP) extracted information from all 15 articles, and the remaining 4 reviewers (DH, SY, YK, and MK) independently extracted information from 3 to 4 articles per person. Discrepancies were reviewed by five authors and resolved in discussion with the sixth author (BK) after re-examining the data to ensure accuracy. The extracted data included the characteristics of the studies (country, design, setting, sample size, participant characteristics, vulnerability factors, and quality assessment), exposure (target season, temperature, and measure), and outcome (mental health outcome and measurement).

Predefined research questions guided the synthesis. We conducted a structured narrative synthesis organized around: (1) characteristics of indoor temperature exposure related to mental health outcomes in older adults, (2) mental health outcomes in older adults related to indoor temperature levels, and (3) factors associated with indoor temperature exposure at varying levels in older adults. Given the heterogeneity in study designs, exposure data formats, and mental health outcome measures, pooling of effect sizes was not possible; therefore, a meta-analysis was not conducted. Instead, we summarized the direction and consistency of associations for each study-outcome pair. To enhance clarity and comparability for the heterogeneous studies, we organized the findings by outcome domain (sleep problems, emotional problems, social interaction problems, and other mental health issues) and further by exposure type (hot vs cold indoor temperature). When studies reported both objective and subjective measurements, results were presented separately, and any discrepancies were noted to highlight methodological variability.

### Quality Assessment in Individual Studies

The National Institutes of Health quality assessment tool for observational cohort and cross-sectional studies [21] was used for quality assessment. This tool evaluates the internal validity of a study by asking 14 questions. The quality of the studies was categorized based on the number of criteria they met: good (>9), fair (5–9), and poor (<5) [22]. Studies were independently scored by three authors (MKP, DH, and SY) using Microsoft Excel. Any discrepancies regarding quality appraisal were resolved in discussion with the fourth author (BK).

## Results

### Study Selection

**Figure 1.**
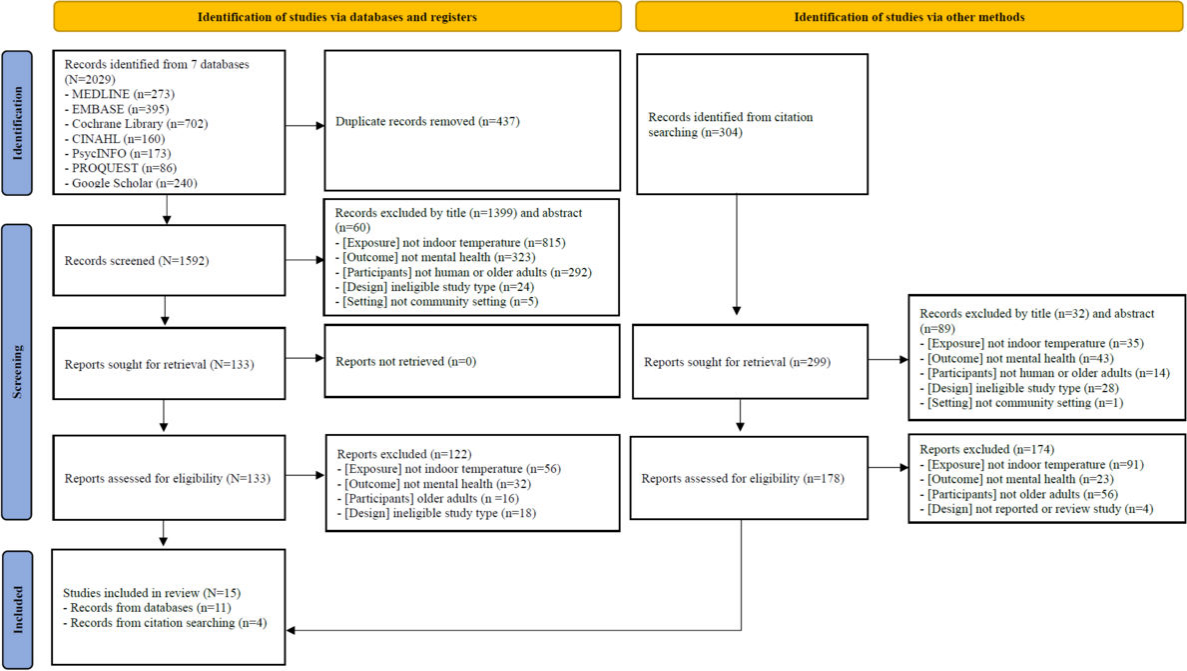
Preferred Reporting Items for Systematic Reviews and Meta-Analyses (PRISMA) flow diagram.

Figure 1 presents the screening and selection process in accordance with PRISMA 2020 guidelines, detailing the number of records included and excluded at each stage, as well as the specific reasons for exclusion during the eligibility assessment. A total of 2328 records were identified through electronic database searches (n=2029) and citation searching (n=299). After removing 437 duplicates, 1891 articles remained. A total of 1580 articles were excluded after title (n=1431) and abstract (n=149) screening for the following reasons—ineligible exposure (not indoor temperature), ineligible outcome (not mental health), ineligible population (not human or not older adults), ineligible study design (eg, editorial or review), and noncommunity setting (eg, hospital)— leaving 311 articles for full-text assessment. Following a detailed eligibility assessment, 296 articles were excluded owing to ineligible exposure, outcome, population, or study design. Ultimately, 15 studies met the inclusion criteria and were included for the final synthesis.

### Characteristics of Included Studies

**Figure 2.**
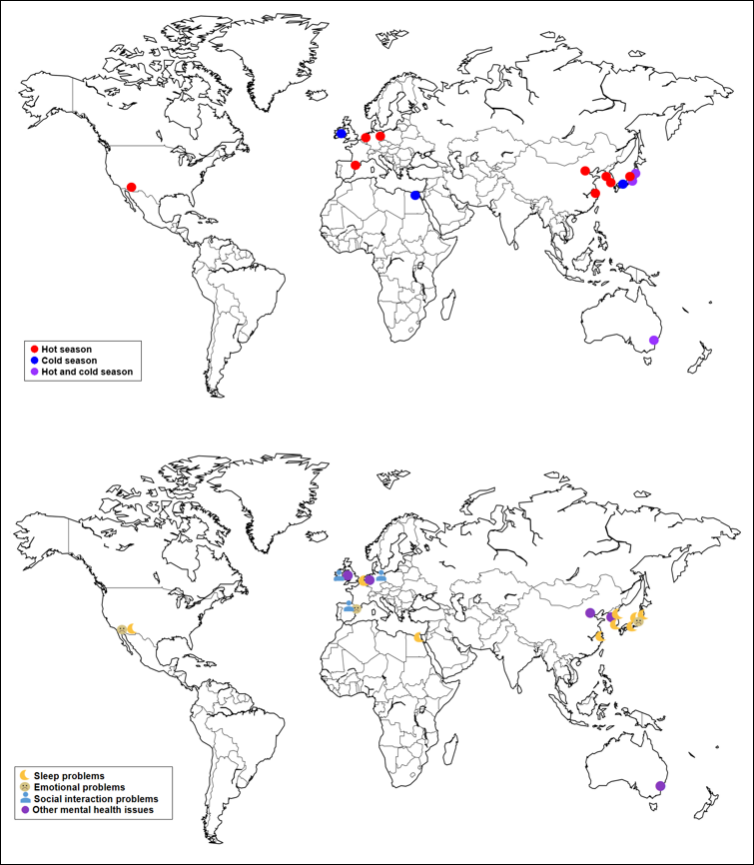
Target season and mental health outcomes by region.

Table 1 presents the characteristics of the included studies. Of the 15 included studies, all except 1 (7%) study [23] were published after 2010, with 10 published within the last 10 years. The majority of the studies (8/15, 53%) were conducted in Asia; the remainder were conducted in Europe (4/15, 27%) and Australia, Egypt, and the United States (1/15, 7% each). As shown in Figure 2, according to the World Bank classification [24], most studies (12/15, 80%) were conducted in high-income countries. From the 15 studies, 7 (47%) used a longitudinal design, whereas the remaining 8 (53%) used a cross-sectional design. Eleven (73%) studies focused on older adults residing at home, and the remaining 4 targeted older adults residing in nursing homes (2/15, 13%), geriatric homes (1/15, 7%), and sheltered facilities (1/15, 7%). The sample sizes ranged from 19 to 29,380. Three (20%) studies included participants with a mean age in the 60s, and 7 (47%) and 2 (13%) studies involved participants with a mean age in the 70s and 80s, respectively. The remaining 3 (20%) studies did not report mean age. Regarding participant characteristics, 3 (20%) studies examined healthy older adults [17,23,25] where 2 (13%) focused on older adults with dementia [26,27], and 1 (7%) examined low-income older adults [28].

**Table 1.** Characteristics of the included studies (N=15).

Authors (year)	Country (urban or rural)	Study design	Setting	Sample size	Age (y), mean (SD; range)^c^	Quality assessment
Ohnaka (1995) [23]	Japan (urban)^a^	Cross-sectional observational study	Home	20	73.0 (67–82)	Good quality
Okamoto-Mizuno and Tsuzuki (2010) [25]	Japan (urban)^a^	Longitudinal observational study	Home	19	65.8 (2.6; ≥62)	Good quality
Bakr et al (2012) [6]	Egypt (urban)^b^	Cross-sectional observational study	Geriatric home	184	68.02 (6.71; 60–97)	Poor quality
Cotter et al (2012) [29]	Ireland (mixed)^b^	Cross-sectional observational study	Home	722	72.5	Poor quality
Garre‐Olmo et al (2012) [26]	Spain (urban)^a^	Cross-sectional observational study	Nursing home	160	82.6 (11.6)	Fair quality
Saeki et al (2015) [30]	Japan (rural)^a^	Longitudinal observational study	Home	861	72.1 (7.1; ≥60)	Good quality
Ahrentzen et al (2016) [28]	USA (urban)^b^	Longitudinal observational study	Home	57	73 (62‐92)	Fair quality
Van Loenhout et al (2016) [31]	Netherlands (urban)^b^	Longitudinal observational study	Home	113	73.8 (7.5; ≥65)	Fair quality
Tartarini et al (2017) [27]	Australia (urban)^a^	Longitudinal observational study	Nursing home	21	(61–92)	Good quality
Kim et al (2020) [32]	Korea (rural)^b^	Longitudinal observational study	Home	104	79.6 (65–96)	Fair quality
Lindemann et al (2018) [33]	Germany (urban)^b^	Longitudinal observational study	Facilities of sheltered	81	80.9 (6.53; 63–93)	Good quality
Lee et al (2020) [34]	Korea (mixed)^a^	Cross-sectional observational study	Home	248	N/A^d^ (≥60)	Fair quality
Kanno et al (2022) [16]	Japan (mixed)^a^	Cross-sectional observational study	Home	29,380	61.7 (11.5)	Fair quality
Yan et al (2022) [17]	China (urban)^a^	Cross-sectional observational study	Home	40	70.7 (5.0)	Good quality
Liu et al (2023) [15]	China (mixed)^b^	Cross-sectional observational study	Home	356	N/A (≥60)	Fair quality

a Participants’ residence was categorized as urban, rural, or mixed based on regional information.

b In the literature, participants’ characteristics are clearly classified as urban or rural, with over 90% sharing the same regional background.

c Age values are presented as reported; mean, SD, and/or range are provided only when available.

d N/A: not applicable.

### Quality Assessment in Individual Studies

The risk of bias assessment for the 15 studies is summarized in Table 1. The quality of the included studies varied. Six (40%) studies were rated as having “good quality.” Seven (47%) studies were categorized as “fair quality” and 2 (13%) as “poor quality.” Bias was primarily due to the unclear participation rate of eligible persons, a lack of justification for the sample size, and the inability to blind the exposure. Detailed results from the quality assessment of each study are available in Table S2 in Multimedia Appendix 2.

### Characteristics of Indoor Temperature Exposure

The seasonal background of the studies was predominantly hot (9/15, %), with 3 (20%) targeting the cold season and 3 (20%) including both hot and cold seasons. The characteristics of indoor temperatures to which older adults were exposed are shown in Table 2. The results for indoor temperatures were presented as means in 10 studies, of which 3 (20%) also reported maximum and minimum temperatures. One (7%) study provided the set air conditioning temperature, while 5 (33%) studies did not specify the exact temperatures. The average indoor temperature during the hot season ranged from 22.7 °C to 30.5 °C, whereas the average temperature during the cold season ranged from 9.5 °C to 17.1 °C.

**Table 2.** Characteristics of indoor temperature (N=15).

Authors (year)	Season and time ▪ Target season (hot or cold) ▪ Month and year	Exposure measure ▪ Objective versus subjective ▪ Location ▪ Duration/interval ▪ Measurement tool	Exposure temperature ▪ Format of temperature reporting ▪ TMean^a^ (SD^b^; TRange^c^)	Vulnerability characteristics ▪ Vulnerable group ▪ Related indoor temperature
Ohnaka et al (1995) [23]	▪ Hot ▪ July–August (year N/A^d^)	▪ Objective ▪ Home (bedroom) ▪ July to August or every 2 minutes throughout the night ▪ Data logger (XT-102, JNIS)	▪ TMean (SD; TRange) ▪ 26.26 °C (2.24 °C; 25 °C-28 °C)	▪ Younger age ▪ Higher room temperature
Okamoto-Mizuno and Tsuzuki (2010) [25]	▪ Cold and hot ▪ February, July–August, October–November (year N/A)	▪ Objective ▪ Home (bedroom) ▪ 5 days or every 1 minute. ▪ Thermistor and hygrometer probe (RS-12; Espec Mic Corporation)	▪ TMean (SD) ▪ Fall: 15.4 °C (0.25 °C) Winter: 9.5 °C (0.69 °C) Summer: 27.7 °C (0.63 °C)	N/A
Bakr et al. (2012) [6]	▪ Cold ▪ October–December (2010)	▪ Subjective ▪ N/A ▪ N/A ▪ Self-reported questionnaire	▪ Response rate (%): feeling cold or hot ▪ N/A	N/A
Cotter et al (2012) [29]	▪ Cold ▪ January–April (2011)	▪ Subjective ▪ Home ▪ N/A ▪ Self-reported questionnaire	▪ Response rate (%) ▪ N/A	▪ Poorer health condition (disability, arthritis, and fall in the previous 6 mo); poorer quality housing (mold, damp, and draughts in the home; no central heating system); difficulty paying for heating; social exclusion (feeling loneliness; low social activities or hobbies) ▪ Very cold homes
Garre‐Olmo et al (2012) [26]	▪ Hot ▪ April–July (2008)	▪ Objective ▪ Home (bedroom, dining room, and living room) ▪ 7 days or twice in the morning and twice in the afternoon ▪ DT-8820 environment meter	▪ TMean (SD), TMedian^e^ ▪ 25.8 °C (1.3 °C)	N/A
Saeki et al (2015) [30]	▪ Cold ▪ October–April (2010‐2013)	▪ Objective ▪ Home (living room and bedroom) ▪ 2 days or every 10 minutes ▪ Thermochron iButtons (DS1922L; Maxim Integrated)	▪ TMean ▪ Evening 17.1 °C (4.1 °C)	N/A
Ahrentzen et al (2016) [28]	▪ Hot ▪ June–August (2010‐2012)	▪ Objective ▪ Home (kitchen, bedroom, and living area) ▪ 5 days or every 15 minutes ▪ Mobile Onset HOBO data loggers	▪ TMean (SD), TMedian, TMax^f^, TMin^g^, TRange ▪ Panel 1: 26.0 °C (1.31 °C; 24.2 °C‐28.0 °C) ▪ Panel 3: 25.4 °C (1.27 °C; 22.9 °C‐28.2 °C)	▪ Affordable housing (low energy efficiency housing) ▪ Higher indoor temperature (mean minimum and exceed 27 °C)
Van Loenhout et al (2016) [31]	▪ Hot ▪ August (2012)	▪ Objective ▪ Living room and bedroom ▪ 21 days with high temperature and 1 cold reference week or every 30 minutes ▪ iButton Hygrochron temperature or humidity loggers (type DS1923) ▪ Subjective ▪ Living room and bedroom ▪ N/A ▪ Self-reported questionnaire	▪ TMean, TMax, TMin, TRange ▪ Living room: 25.4 °C (22.3 °C‐30.2 °C) Bedroom: 25.1 °C (20.8 °C‐29.3 °C)	N/A
Tartarini et al (2017) [27]	▪ Cold and hot ▪ March–December (2015)	▪ Objective ▪ Nursing home (each room) ▪ 14 days or every 15 minutes ▪ iButton temperature loggers (Maxim Integrated)	▪ TMean, TRange ▪ 23.5 °C (16.2 °C‐33.6 °C)	N/A
Kim et al (2020) [32]	▪ Hot ▪ August (2018)	▪ Objective ▪ Home ▪ 3 days or twice a day (morning and afternoon) ▪ Electronic hygrothermographs (AE-817CE, B&J)	▪ TMean, TMax, TMin, TRange, with or without air conditioners ▪ 30.5 °C (22.9 °C‐38.3 °C)	N/A
Lindemann et al (2018) [33]	▪ Hot ▪ May–October (2015) (including heatwave period)	▪ Objective ▪ Facility (table in the shade in the room) ▪ From May to October and 2 heat waves in July and August or every 4 weeks ▪ Data logger (HL-1D, ROTRONIC Messgere GmbH)	▪ TMean, increased temperature ▪ 22.7 °C	▪ Lower gait speed; living in city or city-center (lower access to green spaces compared to living in garden cities or suburban) ▪ Stronger mean changes of participation per 10 °C increase of indoor temperature
Lee et al (2020) [34]	▪ Hot ▪ Heatwave, 2018 (survey conducted in September 2018)	▪ Objective ▪ Home and primary living spaces other than home (eg, workplace and school) ▪ N/A ▪ N/A	▪ Use air conditioner set ▪ Lower than 24 ℃	N/A
Kanno et al (2022) [16]	▪ Cold and hot ▪ May (2013)–November (2018)	▪ Subjective ▪ Home (living room, bedroom, sanitary space, toilet, and corridor or stairs) ▪ N/A ▪ Self-reported questionnaire (CASBEE^h^ Housing Health Checklist related to coldness or warmth)	▪ Evaluation scores ▪ N/A	▪ Younger age; with psychological distress; sleeping difficulties ▪ Poorer housing condition related to coldness or warmth
Yan et al (2022) [17]	▪ Hot ▪ July–August (2018)	▪ Objective ▪ Bedroom ▪ 6 days or every 5 minutes ▪ Data logger (TR-76Ui logger)	▪ TMean, TRange ▪ 28.8 °C (26 °C‐32 °C)	N/A
Liu et al (2023) [15]	▪ Hot ▪ October (2021)–March (2022)	▪ Subjective ▪ Home ▪ N/A ▪ Self-reported questionnaire (8 items about temperature and humidity)	▪ Evaluation score ▪ N/A	N/A

a TMean: mean temperature.

b SD

c TRange: temperature range.

d N/A: not applicable.

e TMedian: median temperature.

f TMax: maximum temperature.

g TMin: minimum temperature.

h CASBEE: comprehensive assessment system for building environment efficiency.

The methods used to measure exposure to indoor temperatures varied significantly across the studies. From the 15 studies, 10 (67%) objectively assessed indoor temperature exposure using devices such as data loggers, while 4 (27%) studies used only self-report questionnaires for subjective evaluation. Among the studies conducted during the cold season, only 1 (7%) [30] objectively assessed indoor temperature exposure. The location of indoor temperature measurements also differed, with bedrooms being the most frequently measured area (8/15, 53%), followed by living rooms (5/15, 33%). Regarding the duration and frequency of temperature measurements, 10 (67%) studies provided this information. The duration of exposure measurements across the studies varied from a few days to several months. Continuous measurements at intervals of 1 to 30 minutes were conducted in 7 (47%) studies, twice a day in 2 (13%) studies, and every 4 weeks in 1 (7%) study.

### Mental Health Outcomes Related to Indoor Temperature Exposure

Table 3 shows that mental health outcomes related to indoor temperature exposure were disproportionately represented by sleep-related outcomes, with 9 of the 15 (60%) included studies focusing on this domain. Sleep problems, including short sleep duration, sleep disturbances, sleep disorders, and insomnia, were negatively associated with both hot and cold indoor temperature exposure. This predominance of sleep-related findings underscores existing gaps in the literature, as relatively few studies have examined other mental health outcomes such as mood, cognition, or social-emotional well-being in relation to indoor temperature. Of the 15 total studies, only 4 (27%) used objective methods to focus exclusively on sleep problems [17,23,25,30], whereas the remaining 11 (73%) studies measured outcomes using subjective methods. In the study by Okamoto-Mizuno and Tsuzuki [25], the association between hot indoor temperature exposure and objective sleep disturbance was statistically significant—wake episode (*r*=0.46; *P*<.05), sleep efficiency index (*r*=−0.47; *P*<.05), and wake after sleep onset (*r*=0.49; *P*<.01)—whereas the association with subjective sleep disturbance was not (*P*<.05). This highlights the differences between subjective and objective outcomes. The findings regarding the association between hot or cold indoor temperature exposure and emotional problems were inconsistent. In the studies by Ahrentzen et al [28] and Kanno et al [16], significant differences in emotional distress (*t*=−2.085; *P*<.05) and psychological distress (*P*<.001) scores were observed based on indoor temperature exposure. By contrast, Garre-Olmo et al [26] did not find indoor temperature exposure to be a significant influence on signs of negative affective mood (*β*=−.10‐.20; *P*>.05).

**Table 3.** Mental health outcomes related to indoor temperature (N=15).

Authors (year)	Mental health outcomes				Outcome measure measurement tool
	Sleep problems	Emotional problems	Social interaction problems	Other mental health issues	
Ohnaka (1995) [23]	Body movement during sleep (*r*=0.299; *P*<.01)	N/A^a^	N/A	N/A	Objective: using a static charge sensitive bed
Okamoto-Mizuno and Tsuzuki (2010) [25]	Objective: sleep disturbance^b^ wake episode (*r*=0.46*; P*<.05) sleep efficiency index (*r*=-0.47; *P*<.05) wake after sleep onset (*r*=0.49; *P*<.01) Subjective sleep disturbance (*r*=not described; *P*>.05)	N/A	N/A	N/A	Objective: using wrist actigraphy Subjective: self-reported questionnaire
Bakr et al (2012) [6]	Insomnia (*P*=.02)	N/A	N/A	N/A	Subjective: self-reported questionnaire (Athens Insomnia Scale)
Cotter et al (2012) [29]	N/A	N/A	Social exclusion (frequency =21.4%; *P*=not applicable)	Loneliness (frequency =26.4%; *P*=not applicable)	Subjective: self-reported questionnaire
Garre‐Olmo et al (2012) [26]	N/A	Signs of negative affective mood (*β*=–.10‐0.20; *P*>.05)	Behavioral signs of social interaction (*β*=–.03 to 0.18; *P*>.05)	N/A	Subjective: proxy-response questionnaire (Quality of Life in Late-Stage Dementia)
Saeki et al (2015) [30]	Objective sleep onset latency (*β*=–.019; *P*=.02) Subjective sleep onset latency (*β*=–.021; *P*<.01)	N/A	N/A	N/A	Objective: using actigraphy Subjective: sleep diary
Ahrentzen et al (2016) [28]	Sleep hours (*t*=2.150, *P*<.05)	Emotional distress (*t*=−2.085; *P*<.05)	N/A	N/A	Subjective: self-reported questionnaire
Van Loenhout et al (2016) [31]	Sleep disturbance (*β*=1.24, *P*=.001)	N/A	N/A	Annoyance (*β*=1.28 to 1.33; *P*<.001)	Subjective: hourly diary
Tartarini et al (2017) [27]	N/A	N/A	N/A	Agitation (*t*=3.09; *P*=.002)	Subjective: informant rating questionnaire (Cohen-Mansfield Agitation Inventory)
Kim et al (2020) [32]	Sleep disturbance (frequency=58.7%; *P*=N/A) Sleep hours (*t*=-0.680; *P*>.05)	N/A	N/A	N/A	Subjective: self-reported questionnaire
Lindemann et al (2018) [33]	N/A	N/A	Social participation (*β*=–4.53; *P*<.05)	N/A	Subjective: self-reported questionnaire
Lee et al (2020) [34]	Sleep disorder^c^ (OR=0.68, *P*>.05)	N/A	N/A	Mental disorder (including depression, anger, anxiety)^c^(OR=1.61, *P*<.05)	Subjective: self-reported questionnaire
Kanno et al (2022) [16]	N/A	▪ Psychological distress (χ2=not described; *P*<.001)	N/A	N/A	Subjective: self-reported questionnaire (Kessler Psychological Distress Scale)
Yan et al (2022) [17]	Objective sleep quality total sleep time (*β*=−86.59, *P*=.02) sleep efficiency (*β*=−7.96, *P*=.01) wake time (*β*=15.37, *P*=.04) REM sleep (*β*=−32.97, *P*=.04) light sleep (*β*=−38.90, *P*=.02) deep sleep (*β*=−27.34, *P*=.27) Subjective sleep quality calmness of sleep (*β*=-1.05, *P*=.02) ease of falling asleep (*β*=-1.25, *P*=.01) ease of awakening (*β*=−0.23, *P*=.26) freshness after awakening (*β*=-1.56, *P*=.02) satisfaction with sleep (*β*=−2.26, *P*=.01)	N/A	N/A	N/A	Objective: using a wrist-worn sleep tracker (Fitbit) Subjective: self-reported questionnaire
Liu et al (2023) [15]	N/A	N/A	N/A	Mental health (*r*=.234, *P* < .01) vitality role limitations due to emotional problems social functioning mental health	Subjective: self-reported questionnaire

a N/A: not applicable.

b Results comparing bedroom temperature in summer and other seasons (fall and winter).

c Results for setting temperature <24 °C.

Similarly, the 3 (20%) studies showed inconsistent findings regarding the association between indoor temperature exposure and social interaction problems. In the study by Lindemann et al [33], exposure to hot indoor temperatures significantly reduced social life participation (*β*=−4.53; *P*<.05) and hindered older adults’ participation in social activities, as reflected in their dissatisfaction with opportunities related to general activity, time management, the amount of activity, and participation in the community. By contrast, Garre-Olmo et al [26] found that indoor temperature was not a significant factor affecting the amount of social interaction. Cotter et al [29] identified a significant link between living in a cold home and social exclusion (21.4% in cold homes vs 17.4% in the total sample). In addition, significant associations were found between exposure to hot or cold indoor temperature and other mental health issues (5/15, 33%), including agitation (*t*=3.09; *P*=.002) [27], loneliness (26.4% in cold homes vs 12% in the total sample) [29], and annoyance (*β*=1.28‐1.33; *P*<.001) [31].

### Factors Associated With Vulnerability to Inappropriate Indoor Temperature Exposure

Five (33%) studies reported characteristics related to vulnerability to exposure to inappropriate indoor temperature. Residents living in poorly conditioned houses, particularly those without energy-efficiency upgrades, were found to be vulnerable to indoor temperature exposure, which can exacerbate health issues, particularly during hot weather [28]. Older adults living in cities or city centers experienced a greater increase in higher indoor temperature exposure than those living in garden cities or suburbs owing to lower access to green spaces [33]. Factors identified as increasing vulnerability to cold indoor temperature exposure included poorer health conditions (eg, disability, arthritis, and fall in the previous 6 months), poorer quality housing (eg, mold, damp, and draughts in the home; no central heating system), difficulty in paying for heating, and social exclusion (eg, feeling lonely and low social activities or hobbies) [29]. In addition, lower gait speed [33], younger age [16,23], and being female [16] were associated with greater exposure to hot or cold indoor temperatures.

## Discussion

### Principal Findings

To the best of our knowledge, this is the first systematic literature review investigating the association between exposure to varying indoor temperature levels and mental health among older adults. We found that exposure to both high and low indoor temperatures is associated with mental health problems in older adults. Specifically, sleep, emotional, and social interaction problems are frequently reported in association with indoor temperature levels.

### Indoor Temperature Levels and Mental Health

Sleep disturbances were the most consistently reported mental health issue related to indoor temperature levels. Several studies suggested that both hot and cold indoor environments were associated with poorer sleep quality, including short sleep duration [28], insomnia [6], sleep disturbances [32], sleep disorders [34], and longer sleep-onset latency [30]. This finding is consistent with recent reviews, which have confirmed the negative association between extreme outdoor temperature exposure and sleep [35,36]. Van Loenhout et al [31] reported that more than 40% of older adults experienced severe sleep disturbances when indoor temperatures exceeded 25 °C, even during short heatwaves. The optimal indoor temperature for sleep is influenced by several factors, including regional climate and housing conditions [10,11], ventilation method used [10], and the type of bedding and sleepwear [11]. Therefore, it is important not only to maintain an appropriate bedroom temperature range but also to create a comfortable sleeping environment through adaptation strategies, such as appropriate bedding, sleepwear, and ventilation, to promote sleep health in older adults.

Indoor temperature levels were also reported to be associated with emotional problems, reduced social interaction, and symptoms such as agitation, loneliness, and annoyance in older adults. Regarding emotional problems, evaluations of housing coldness or warmth are significantly related to psychological distress [16]. In terms of social interactions, significant relationships have been identified between living in a cold home, social exclusion, and loneliness [29]. In addition, participation in social life decreases with increasing indoor temperatures [33]. Furthermore, the cumulative exposure to both hot and cold temperatures has been shown to correlate linearly with agitation [27], and increasing indoor temperatures are associated with heightened annoyance due to heat [31]. These findings are consistent with recent studies on outdoor temperature exposure, which have reported that high temperatures are associated with mood disturbances, reduced social participation due to decreased motivation, aggravation of preexisting mental health conditions, and reduced patience and tolerance [37]. However, in this analysis of indoor temperature exposure, the number of studies on outcomes other than sleep problems was three or fewer, and the methods of reporting results and statistical significance varied among studies, limiting the conclusions that can be drawn about the impact.

Meanwhile, we determined that there is a lack of studies investigating the impact of indoor temperature levels on depression, suicide, and anxiety, which have been highlighted as highly associated with outdoor temperatures [38,39]. To address these limitations, there is a need for longitudinal cohort studies and randomized intervention trials that can more robustly evaluate the causal relationships between indoor temperature exposure and a wider range of mental health outcomes in older adults.

### Vulnerable Groups’ Exposure to Inappropriate Indoor Temperature

Most of the characteristics related to inadequate indoor temperature exposure identified in this study were vulnerability-modifying factors that either amplified or mitigated the association between exposure and mental health outcomes. For example, this review demonstrates how poor housing and low economic status affect the mental health of older adults through indoor temperature exposures. Older adults living in affordable housing with low energy efficiency [28] or facing economic difficulties such as constraints in paying heating bills [29] are exposed to hotter indoor environments in summer and colder ones in winter. This exposure is related to various mental health outcomes such as emotional distress, reduced sleep length and quality, loneliness, and social exclusion. Older adults are considered the group most affected by poor housing conditions and are also the age group most likely to reside in such conditions [40]. Managing residential environments can be an effective strategy for improving the health outcomes of vulnerable populations by enhancing thermal comfort [41].

In addition, older adults living in cities or city centers with lower access to green spaces were more vulnerable to changes in social participation due to rising temperatures than those living in garden cities or suburban areas [33]. This might have been owing to the urban heat island effect. The urban heat island effect associated with urbanization can cause temperatures within cities to be significantly higher than those in surrounding rural areas, which is exacerbated when there is a lack of green spaces [42]. Previous studies have also emphasized that older adults living in urban areas are at the greatest risk during heatwaves [43]. As the aging population increasingly moves to cities for health care access [44], addressing the challenges of urban heat by improving infrastructure and integrating green spaces will be essential to protecting the well-being of older adults.

On the contrary, certain preexisting conditions, such as disabilities or psychological distress, may act as potential confounders that influence both indoor temperature exposure and mental health outcomes. Previous studies have shown that people with disabilities experience mental distress 4.6 times more often than do adults without disabilities [45]. At the same time, people with disabilities have a higher risk of heat exposure-related emergency department admissions, particularly for mental disorders, compared with those without disabilities [46]. Therefore, future research should distinguish between vulnerability modifiers and potential confounders for accurate interpretation of the association between indoor temperature and mental health. This distinction will strengthen causal inference and support more precise identification of at-risk groups and intervention priorities.

Policy responses addressing the impact of inadequate indoor temperature exposure on vulnerable groups are limited. Although the World Health Organization’s 2018 Housing and Health Guidelines [47] outline the importance of managing indoor temperatures for mental well-being, few policies focus directly on this issue. For example, while the US Department of Health and Human Services provides heating and cooling assistance for low-income households, these programs lack specific guidelines on optimal indoor temperatures for mental health [48]. Similarly, South Korea’s Health Plan 2030 includes initiatives for creating age-friendly environments but does not focus on indoor temperature management [49]. Therefore, future policies should integrate evidence on the mental health impacts of indoor temperature exposures. From a policy perspective, legal integration could involve the incorporation of standardized indoor temperature thresholds into national building codes and long-term care facility regulations to safeguard the mental health of older adults. Embedding these standards within existing regulatory frameworks would promote compliance and sustainability, while establishing specific implementation guidelines could support nurses and facility managers in adhering to them. In community settings, incorporating indoor temperature assessments into home-visiting nursing protocols—where nurses systematically record environmental conditions alongside vital signs—could enhance surveillance of thermal environments and inform evidence-based interventions. It may be a useful strategy to prioritize older adults with poor health, those living in substandard housing, those with low economic status, and those experiencing social exclusion—identified in this review as being vulnerable to exposure to inappropriate indoor temperatures—as the target population for community home nursing services.

### Measuring Indoor Temperature and Mental Health Outcomes

Objectively measuring indoor temperature exposure and identifying the appropriate temperature ranges and thresholds are the first steps to protecting older adults from extreme temperatures. Compared with younger generations, older adults are less sensitive to thermal stimuli and tend to feel less heat [50]. This means that older adults may feel thermally comfortable at temperatures that in fact could pose health risks. In our review, 6 (40%) studies [6,16,25,27,29,30] investigated sleep problems in cold environments, but only one [30] used objective temperature measurements, making it difficult to determine precise threshold values of safe temperature. The lack of objective data reduces the potential of leveraging behavioral measures to control indoor temperatures. Therefore, further research is needed to establish the optimal indoor temperature conditions for the mental health of older adults. This will enable health care providers to educate older adults on appropriate indoor temperature ranges and help them adjust their indoor temperature exposure.

This study highlights the importance of a balanced approach to using both subjective and objective measures of mental health outcomes to gain a comprehensive understanding of the influence of indoor temperature exposure. Our findings revealed that only sleep-related outcomes were objectively assessed in the literature, whereas other outcomes were measured solely using self-reported questionnaires. While subjective measures capture individual perceptions that can strongly influence behavior, they may introduce bias in older adults with mental health issues or memory limitations, potentially leading to inaccurate results when used alone. Conversely, a study conducted by Okamoto-Mizuno and Tsuzuki [25] revealed that relying solely on objective methods could result in underreporting of sleep disorders, as subjective sleep disturbances are not reflected in objective measurements. Similarly, previous studies have identified discrepancies between subjective and objective sleep assessments in older adults and recommended using both assessment methods for a more balanced evaluation [51]. Therefore, it is crucial to consider objective and standardized methods alongside subjective methods to measure mental health outcomes in older adults. Recently, methods that minimize recall bias by repeatedly measuring participants’ experiences in real time using smartphones have been effectively applied to assess mental health outcomes in older adults [52,53]. Incorporating these innovative methods into research could improve the accuracy of measuring subjective mental health outcomes related to changes in indoor temperature exposure. In addition, linking mental health outcome data to corresponding indoor temperature measurements would provide more comprehensive insights.

### Limitations

This study had several limitations. First, most included studies were conducted in high-income countries, with a notable number in Asia. This may limit the generalizability of the findings to other regions or lower-income settings where the impact of indoor temperature exposure on mental health might differ. Second, the heterogeneity of study designs, settings, and measurement methods in the included studies prevented the possibility of a meta-analysis. Future research using standardized methodologies is needed to establish more robust evidence. Third, the limited diversity of the outcomes in the included studies also limited our ability to fully grasp the wide-ranging impact of indoor temperature exposure on the mental health of older adults, highlighting the need for future research that explores a broader range of mental health outcomes.

### Implications

Despite the limitations, the findings of this study have significant implications for nursing care, education, research, and policy development. In nursing care, assessing indoor temperature exposure during home visits to older adults, particularly those facing economic hardships or living in substandard housing conditions, could be a valuable approach to improved recognition and mitigation of threats to mental health. Providing practical and specific guidance on maintaining optimal indoor temperatures may support the mental health of older adults.

From an educational perspective, this study highlights the importance of developing programs aimed at enhancing climate literacy among nurses and health care professionals. Such programs could help health care providers better understand the impact of indoor temperature fluctuations on the mental health of older adults within the context of climate change. In addition, these programs may improve health care providers’ ability to guide older adults and their families on practical strategies for managing indoor temperatures.

In this research, the findings emphasize the value of establishing standardized study designs and measurement tools to examine the association between indoor temperature exposure and mental health. Consistent criteria for temperature measurement and mental health variables would facilitate robust meta-analyses and provide stronger evidence. In addition, future studies could benefit from adopting collaborative, multidisciplinary approaches involving environmental science, public health, and nursing. Conducting longitudinal studies that combine real-time temperature exposure monitoring with mental health assessments can provide stronger causal evidence.

Policies could focus on retrofitting older homes to enhance insulation and thermal regulation, particularly for low-income households. In addition, community-wide initiatives, such as expanding green spaces in urban areas, could mitigate heat island effects, aligning with public health objectives. Health care providers can contribute to these efforts by sharing evidence-based insights from clinical and community settings with policymakers.

### Conclusion

In this systematic review, we identified an association between exposure to both hot and cold indoor temperatures and a range of mental health outcomes in community-dwelling older adults, with sleep disturbances being the most consistently reported. Evidence on other outcomes—such as depression and anxiety, which have been extensively studied in relation to outdoor temperature exposure—remains limited. Future studies using standardized and longitudinal designs are needed to establish optimal indoor temperature conditions and to inform health policies and practices that better protect the mental health of older adults.

## Supplementary material

10.2196/78257 Multimedia Appendix 1 Search strategies.

10.2196/78257 Multimedia Appendix 2 Quality rating by the National Institutes of Health (NIH) quality assessment tool for observational cohort and cross-sectional studies.

10.2196/78257 Checklist 1 PRISMA checklist.

## Abbreviations

PRISMA: Preferred Reporting Items for Systematic Reviews and Meta-Analyses
