# Supplementary material for: Association of Indoor Temperature Level and Mental Health Among Community-Dwelling Older Adults: Systematic Review
Source: JMIR Public Health Surveill. 2025 Nov 27;11:e78257. doi: 10.2196/78257 (PMC12661602; doi:10.2196/78257)
Supplement: Multimedia Appendix 1 [file publichealth-v11-e78257-s001.docx]

**Supplementary Table 1.** Search Strategies

|  | **Population** | **Outcome** | **Exposure** | |  |
| --- | --- | --- | --- | --- | --- |
|  | **Category 1** | **Category 2** | **Category 3** | **Category 4** |  |
| **Keywords** | aged OR elderly OR elder OR older OR older adults OR old OR senium OR senior OR geriatric OR aging OR old age OR centenarian OR nonagenarian OR octogenarian OR septuagenarian OR sexagenarian OR later life | mental health OR mental well-being OR mental problem OR mental deterioration OR emotions OR emotional OR emotional health OR mental disorder OR psychological OR Mental Health Services OR Mental Illness OR Psychomotor Disorders OR Psychomotor Impairment OR Alzheimers OR Dementia OR Vascular dementia OR cognitive decline OR cognitive function OR cognitive dysfunction OR cognitive impairment OR cognitive disorder OR cognitive symptom OR cognitive deficits OR cognitive problem OR memory disorders OR memory decline OR memory deficits OR memory loss OR SCD OR mild neurocognitive disorder OR pre-clinical dementia OR preclinical dementia OR predementia OR Mood Disorders OR Bipolar Disorder OR Depression OR Depressive Disorder OR depressive syndrome OR Melancholia OR Mania OR Manic episodes OR Manic State OR Hypomania OR Hypomanic Episode OR Bipolar and Related Disorders OR Anxiety OR Angst OR Anxiousness OR Hypervigilance OR Nervousness OR Anxiety Disorders OR Phobia OR Phobia, Social OR Personality Disorders OR behavioural disorders OR Emotional Exhaustion OR Emotional Fatigue Delirium OR confusion OR inattentiveness OR disorientation OR illusions OR hallucination OR agitation OR nervous OR delusional OR schizophrenia OR Schizoaffective OR Persistent delusional disorders OR Psychotic Disorders OR Affective Disorders OR Acute psychosis OR Obsessive compulsive disorder OR Alcohol dependence OR substance misuse OR opiate dependence OR cocaine OR amphetamine OR benzodiazepine OR legal highs OR novel psychoactive agents OR Stress OR Somatoform Disorders OR Somatization Disorder OR Neurotic disorders OR Eating Disorders OR Sleeping disorders OR Suicide OR Attempted suicide OR Self-harm OR Overdose OR Panic OR Post-traumatic stress disorder OR Neurotic OR Learning disability OR mental retardation OR autism OR Aspergers OR autistic spectrum disorders | indoor OR home OR residential OR house | temperature OR hot OR heat OR warm OR cold OR hyperthermia, Induced OR hypothermia, Induced OR thermal environment |  |
| **MeSH** | Aged Aged, 80 and over Aging  Geriatrics | Mental health |  | Temperature Hot temperature Cold temperature |  |
|  | **(Category 1) AND (Category 2) AND (Category 3) AND (Category 4)** | | | | |
